# HMGA1/E2F1 axis and NFkB pathways regulate LPS progression and trabectedin resistance

**DOI:** 10.1038/s41388-018-0394-x

**Published:** 2018-07-06

**Authors:** Rossella Loria, Valentina Laquintana, Giulia Bon, Daniela Trisciuoglio, Roberta Frapolli, Renato Covello, Carla Azzurra Amoreo, Virginia Ferraresi, Carmine Zoccali, Mariangela Novello, Donatella Del Bufalo, Michele Milella, Roberto Biagini, Maurizio D’Incalci, Rita Falcioni

**Affiliations:** 1 Cellular Network and Molecular Therapeutic Target Unit, IRCCS Regina Elena National Cancer Institute, Via Elio Chianesi 53, 00144 Roma, Italy; 2 Preclinical Models and New Therapeutic Agents Unit, IRCCS Regina Elena National Cancer Institute, Via Elio Chianesi 53, 00144 Roma, Italy; 3 Institute of Molecular Biology and Pathology, CNR National Research Council, c/o Sapienza University of Rome, 00185 Rome, Italy; 4 Department of Oncology, IRCCS-Istituto di Ricerche Farmacologiche Mario Negri, Via La Masa 19, Milan, Italy; 5 Pathology Unit, Department of Research Advanced Diagnostic and Technological Innovation, IRCCS Regina Elena National Cancer Institute, Via Elio Chianesi 53, 00144 Roma, Italy; 6 Medical Oncology A, IRCCS Regina Elena National Cancer Institute, Via Elio Chianesi 53, 00144 Roma, Italy; 7 Orthopedic Surgery, Department of Experimental Clinical Oncology, IRCCS Regina Elena National Cancer Institute, Via Elio Chianesi 53, 00144 Roma, Italy

## Abstract

Although the medical treatments of sarcoma have evolved in the last years, a significant portion of patients develops recurrence after therapies suggesting the need to identify novel targets to improve the treatments. By the use of patient-derived and established cell lines from liposarcoma, as well as specimens from patient biopsies, we found that HMGA1 is involved in the progression of dedifferentiated and myxoid liposarcoma. The immunohistochemical and RT-PCR analyses of 68 liposarcoma specimens revealed a significant high expression of HMGA1, at the protein and RNA levels, both in myxoid and dedifferentiated liposarcoma subtypes compared with differentiated ones. Loss- and gain-of-function experiments by HMGA1-specific depletion and overexpression in dedifferentiated and myxoid liposarcoma cells showed the contribution of this oncogenic factor in cell proliferation, motility, invasion, and drug resistance. The in vitro and in vivo treatment of myxoid liposarcoma with trabectedin, a drug with a potent anti-tumor activity, revealed downregulation of HMGA1, E2F1, and its-downstream targets, vimentin and ZEB1, indicating a critical role of trabectedin in inhibiting the mesenchymal markers of these tumors through the HMGA1/E2F1 axis. These data were also confirmed in patients’ tumor biopsies being HMGA1, E2F1, and vimentin expression significantly reduced upon trabectedin therapy, administered as neo-adjuvant chemotherapy. Furthermore, trabectedin treatment inhibits in vitro NFkB pathway in mixoyd liposarcoma sensitive but not in resistant counterparts, and the inhibition of NFkB pathway re-sensitizes the resistant cells to trabectedin treatment. These data support the rational for combining NFkB inhibitors with trabectedin in liposarcoma patients, who have become resistant to the drug.

## Introduction

Liposarcoma (LPS) is the most common amongst soft tissue sarcomas (STS), a complex and heterogeneous group of more than 50 neoplasms arising from mesenchymal cells. LPS accounts for 45% of retroperitoneal tumors and 24% of limbs tumors [1]. LPS themselves are heterogeneous adipocyte tumors and are morphologically classified into four subtypes: well differentiated LPS (WDLS), dedifferentiated LPS (DDLS), myxoid LPS (MLS), and pleomorphic LPS (PLS) [2]. Different genetic alterations characterize the different subtypes. Amplification of the chromosome segment 12q13–15, which carries the oncogenes *MDM2*, CDK4, and HMGA2, is found in WDLS and DDLS [3]; translocation of the genes *FUS* and *DDIT3* (*CHOP*) genes are present in MLS [4], while loss of p53 and Rb oncosoppressors characterizes PLS and causes karyotype disorders [5].

Surgical resection with negative margins remains the pivotal treatment of localized LPS [6] though radiotherapy is extensively used in the treatment of sarcoma [7].

Trabectedin (Ecteinascidin-743 or ET-743), a marine alkaloid isolated from the tunicate Ecteinascidia turbinata, has a potent antitumor activity in a wide range of tumors [8–12]. It was approved in Europe and several other countries for the treatment of advanced, metastatic STS no-longer responding to anthracycline-based chemotherapy, and in US for the second line treatment of leyomiosarcomas and LPSs [10–12]. Trabectedin interferes with several transcription factors binding the minor groove of DNA [13, 14]. Specifically it alters chromatin structure and enhancing or inhibiting the activity of several transcription factors regulates the expression of several proteins [15, 16]. It has been demonstrated that trabectedin downregulates abnormal transcription factor expression, such as the rearranged genes *FUS-CHOP* or *EWS-CHOP* that characterize MLS, and modulates the production of cytokines and chemokines causing a profound alteration of tumor microenvironment [17, 18]. In addition, trabectedin impairs the function of the High Mobility Group A (HMGA) proteins reducing the binding to their responsive promoters. This mechanism is believed to be relevant for drug activity as in some cell lines it is influenced by expression of HMGA [19]. Previous studies identified HMGA1-oncogene as a key transcription factor enriched in human embryonic stem (ES) cells, and adult stem cells [20, 21]. The expression of HMGA1 was correlated with the tumor aggressiveness, low level of differentiation, resistance to therapies and poor prognosis in the majority of epithelial tumors [22]. The *HMGA1* gene encodes the low molecular weight HMGA1a and HMGA1b chromatin remodeling proteins, which bind the minor groove of AT-rich DNA sequences [23]. HMGA proteins do not possess transcriptional activating domain, but form multiple protein complexes that, altering chromatin structure and orchestrating the assembly of transcription factor complexes, regulate the transcription of several genes [24–26]. Rearrangements of the HMGA1 gene are present in benign adipocyte tumors characterized by 6p21 chromosome aberrations [27, 28], suggesting a role in fusion transcript-mediated LPS progression.

Here, we evaluated whether HMGA1 plays a role in specific LPS subtypes and contributes to LPS response/resistance to trabectedin treatment.

## Results

### In vivo HMGA1 expression is higher in DDLS and MLS than in DLPS

**Fig. 1:**
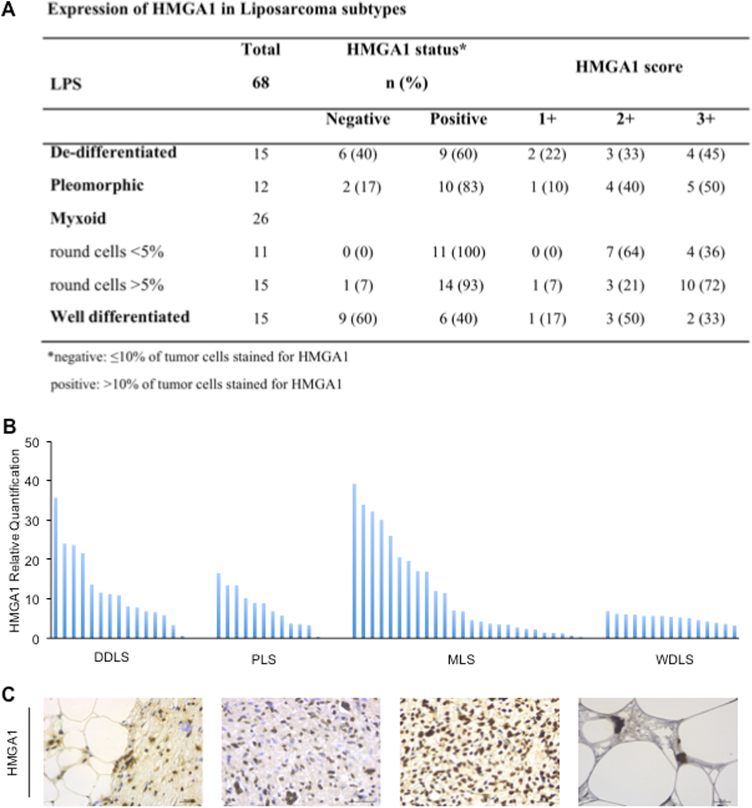
Expression of HMGA1 on a cohort of 68 patients. **a** The cohort of 68 cases of LPS were classified for subtype (DDLS, PLS, MLS, and WDLS), HMGA1 status and score of positivity quantified by IHC analysis. **b** qRT-PCR for HMGA1 was performed on mRNA extracted from sections of liposarcoma specimens. **c** Representative HMGA1 IHC analysis on sections from samples [40×]. Scale bar = 30 µm

In order to evaluate the HMGA1 expression in LPS subtypes, we performed RT-PCR and IHC analyses of LPS specimens derived from a cohort of 68 patients surgically treated at the Regina Elena National Cancer Institute. The pathologist confirmed, by RT-PCR and Fish analyses, the amplification, the loss and the re-arrangement of genes that characterize the karyotype disorders of all LPS included in this study. As reported in Fig. 1a, we analyzed 15 WDLS, 15 DDLS, 26 MLS, and 12 PLS. We showed for the first time that 100% of MLSs were highly positive for HMGA1 expression (score 2+/3+), 60% of DDLS (score 1+/3+), 83% of pleomorphic (score 1+/3+), while only 40% of WDLS was positive for HMGA1 (score 1+/3+) (Fig. 1a). RT-PCR analysis confirmed the IHC data of DDLS and MLS expressing significantly higher level of HMGA1 mRNA (Fig. 1b) than WDLS (*P* < 0.01), suggesting a role of the protein in the mechanism of tumor progression of in the highly aggressive and more de-differentiated LPS subtypes. Though PLS were positive for HMGA1 protein expression, the mRNA levels were not significantly different from those found in WDLS (*P* < 0.15) suggesting a regulation of the protein at the post-transcriptional level/s. Figure 1c shows the IHC analysis of representative HMGA1 positive specimens [40×].

### HMGA1 is involved in cell proliferation and invasion of LPS cells

**Fig. 2:**
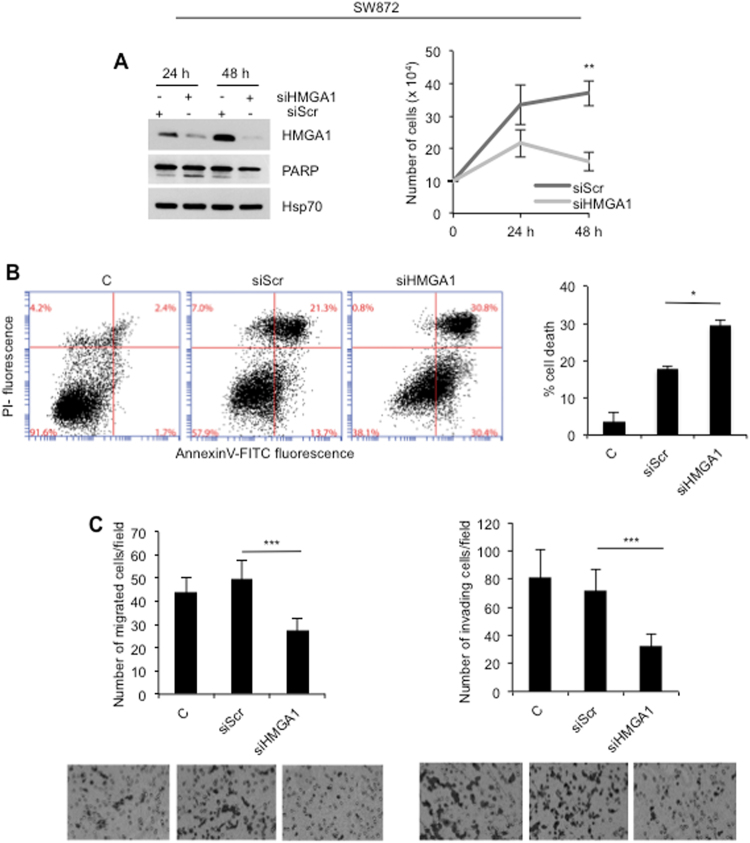
Interference with HMGA1 inhibits cell proliferation, induces cell death, and impairs in vitro motility and invasion of SW872 liposarcoma cells. **a** Total cell lysates from SW872 cells were analyzed 24 an 48 h upon depletion for the expression of HMGA1, PARP, and Hsp70. Cell viability was evaluated by Trypan blue exclusion from three independent experiments (*P* < 0.001). **b** Apoptosis was evaluated by Annexin-V/PI flow cytometric assay. Cell death was presented as the means ± SD of triplicate experiments (*P* < 0.01). **c** Chemotaxis and chemoinvasion assays were performed to examine the effect of HMGA1 depletion on SW872 cells. Number of migrated and invading cells was presented as the means ± SD of triplicate experiments of each group (*P* < 0.0001)

We first analyzed the expression of HMGA1 in LPS cell line SW872. The results revealed high expression of HMGA1 protein in these cells whose levels were comparable with those found in the thyroid tumor cells 8305C used as positive control (Fig. S1); Human fibroblasts (HF) were used as low-expressing control. The expression level of protein correlated with mRNA in each cell line (Fig. S1). To study the function of HMGA1 in SW872 LPS cells, we interfered with the expression of HMGA1 protein by specific siRNA (Fig. 2a). Upon interference, we found that loss of HMGA1 protein in LPS cells causes a strong inhibition of cell proliferation between 24 and 48 h posttransfection (Fig. 2a, right panel). The cleavage of PARP (Fig. 2a, left panel) was indicative of apoptotic cell death, confirmed by FACS analysis (Fig. 2b). Si-RNA-transfection itself caused toxicity, as indicated by the fact that scramble sequence induced some degree of apoptotic cell death not seen in the un-transfected cells (Fig. 2b). In spite of this background noise, downregulation of HMGA1 by specific siRNA induced a statistically significant increase of cell death compared with siScr-transfected cells (*P* < 0.01) (Fig. 2b). HMGA1 has been involved in motility and invasion processes [22]; thus, we analyzed the capability of SW872 cells to move after HMGA1-specific interference. As shown in Fig. 2c, significant reduction of motility and invasion were observed in the HMGA1-interfered cells compared with control (*P* < 0.0001). Of relevance, interference of SW872 cells with a second HMGA1-specific siRNA confirmed the results (Fig. S2).

**Fig. 3:**
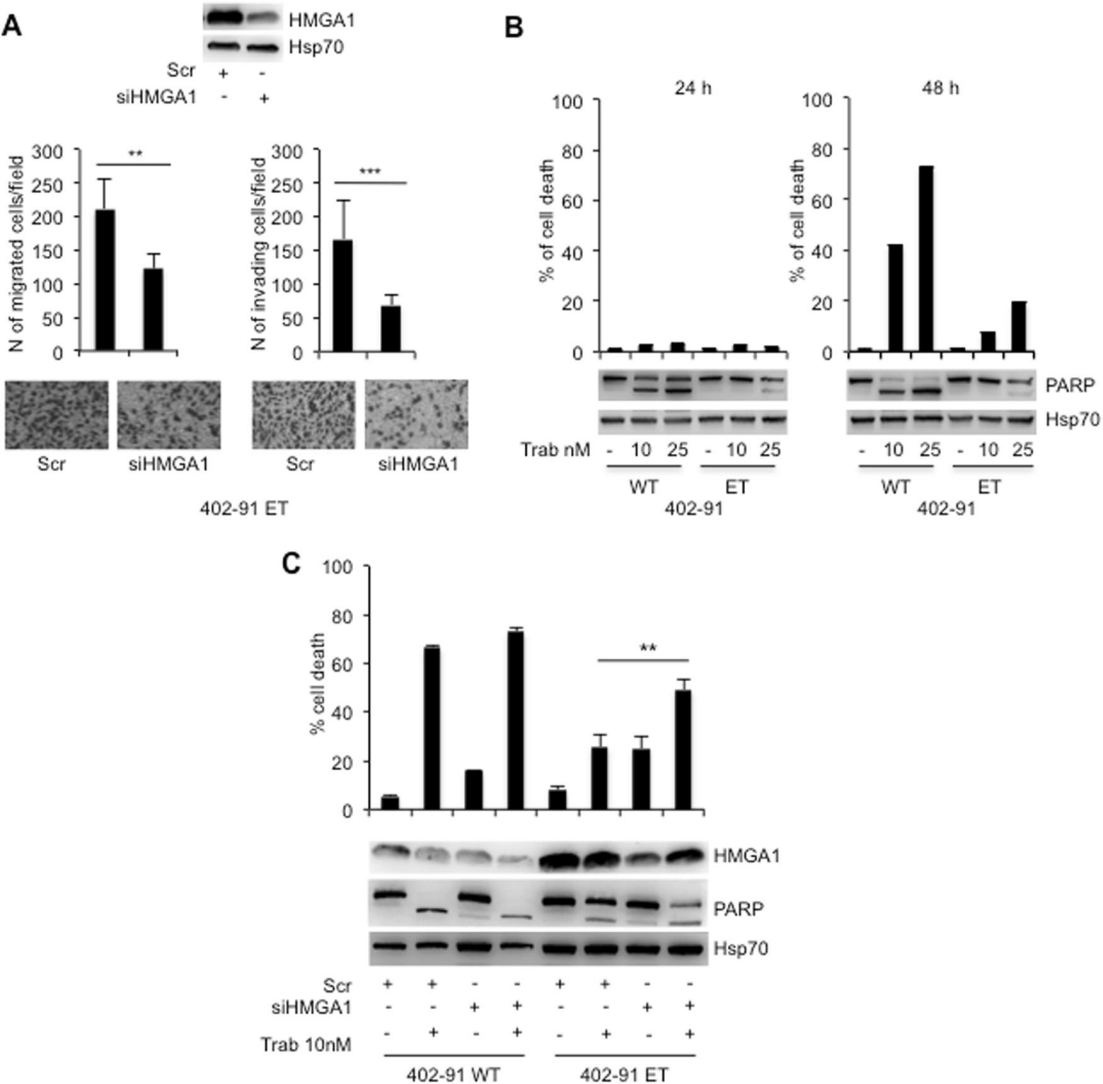
HMGA1 depletion in MLS trabectedin-resistant cells inhibits cell migration and invasion and favors responsiveness to the drug. **a** Chemotaxis and chemoinvasion assays were performed to examine the effect of HMGA1 depletion on 402-91 ET cells. Number of migrated and invading cells was presented as the means ± SD of triplicate experiments of each group (*P* < 0.001 and *P* < 0.0001, respectively). **b** Cell death of 402-91 WT and 402-91 ET cells, upon trabectedin treatment for the indicated time, were evaluated by Trypan blue exclusion from three independent experiments (*P* < 0.0001) (upper panel); total cell lysates were evaluated by WB for the expression of PARP and Hsp70 (lower panel). **c** 402-91 WT and 402-91 ET cells, upon depletion with HMGA1 expression, were treated for 48 h with trabectedin. Total cell lysates, derived from siScr and siHMGA1 untreated or treated cells were analyzed by WB for the expression of HMGA1, PARP, and Hsp70 (lower panel). Cell death was evaluated by Trypan blue exclusion from three independent experiments (*P* < 0.001) (upper panel)

To further evaluate the role of HMGA1 in motility and invasion of LPS, we took advantage from MLS cell line, the 402-91 WT expressing low level of HMGA1 compared with its counterpart 402-91 ET, made resistant to trabectedin treatment (Fig. S3A). We stably transfected both cell lines with an expression vector carrying HMGA1 cDNA (Fig. S3A). The overexpression of HMGA1 in parental 402-91 WT cells conferred a significant induction of their capability to move and invade in vitro compared with un-transfected 402-91 WT cells (Fig. S3B, and C) (*P* < 0.0001). The overexpression of HMGA1 in trabectedin-resistant 402-91 ET cells did not influence their motility and invasion ability (Fig. S3B, C). On the contrary, the interference of HMGA1 in resistant cells significantly reduced both cell motility and invasion (*P* < 0.001 and *P* < 0.0001, respectively) (Fig. 3a). Trabectedin treatment of LPS cells, as expected, induced cell death in the sensitive 402-91 WT cells, reaching 70% upon 48 h of treatment, whereas about 20% cell death was found in the resistant 402-91 ET cells (Fig. 3b). The cleavage of PARP suggested cell death by apoptosis, as previously described [29]; this data were confirmed by FACS analysis showing, after 48 h of treatment, 80% of cell death by apoptosis only in the sensitive cells (Fig. S4). Interestingly, HMGA1 depletion in combination with trabectedin treatment showed additive effect on the cell death (Fig. 3c) of 402-91 ET cells (*P* < 0.001) but not of the sensitive one, suggesting that HMGA1 is involved in the mechanism of trabectedin resistance, at least in MLS (Fig. 3c). In agreement, trabectedin treatment induces downregulation of HMGA1 in 402-91 WT sensitive cells but not in the resistant counterpart (Fig. 3c).

### Trabectedin downregulates HMGA1 and E2F1 and inhibits the mesenchyme markers in vitro and in vivo

**Fig. 4:**
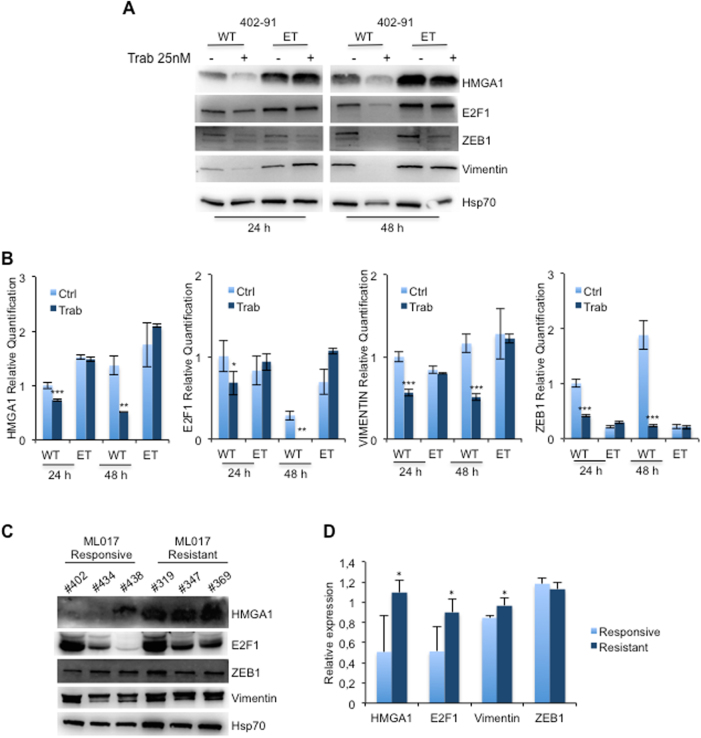
Trabectedin regulates mesenchymal markers expression in vitro and in vivo through HMGA1/E2F1 axis. **a** Total cell lysates and **b** mRNA levels from 402-91 WT and 402-91 ET cells were analyzed by WB and qRT-CR for the expression of HMGA1, E2F1, ZEB1, and Vimentin before and after trabectedin treatment. **c** Total cell lysates from ML017 PDX, responsive (#402, #434, and #438) and resistant (#319, #347, and #369) to trabectedin, were analyzed by WB for the expression of HMGA1, E2F1, ZEB1, and Vimentin. **d** Relative expression was quantified by ImageJ 1.47v using Hsp70 protein for normalization

**Fig. 5:**
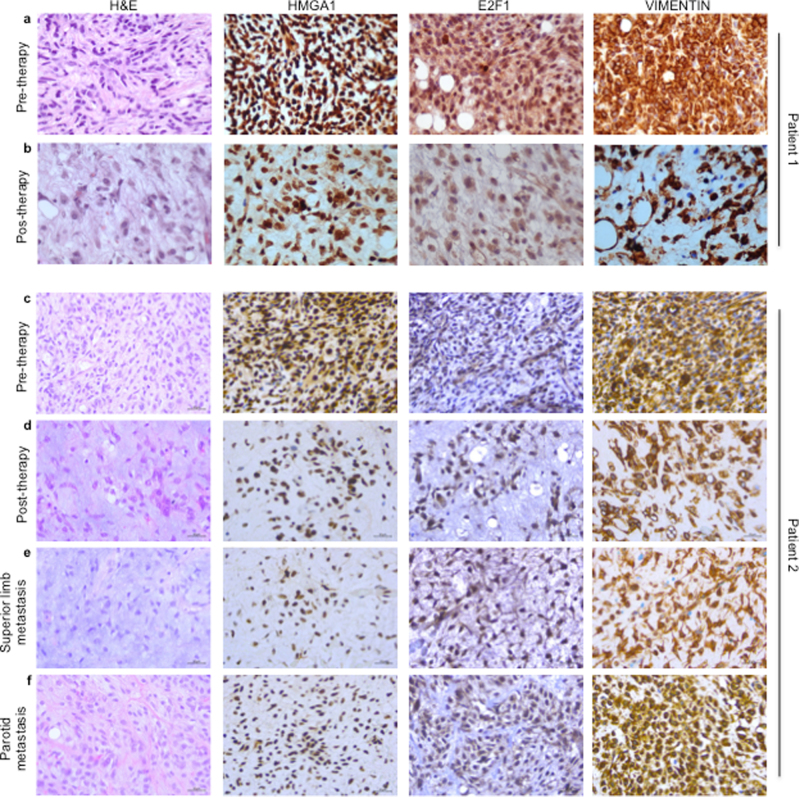
Trabectedin downregulates in vivo mesenchymal markers in MLS after neo-adjuvant chemotherapy. H&E sections and IHC analysis of HMGA1, E2F1, and vimentin in the bioptic sample of MLS tissues before therapy (**a** and **c** panels) and on the surgical sample obtained after trabectedin therapy (**b** and **d** panels). H&E sections and IHC analysis for the expression of HMGA1, E2F1, and vimentin of specimens derived from distant metastases of patient c whose tumor has progressed: superior limb metastasis (**e**), and parotid metastasis (**f**). (Scale bar = 30 μm)

It has been previously reported that HMGA1 protein may have a role during epithelial to mesenchymal transition (EMT) through the transcriptional regulation of several target genes that maintain the EMT condition [30, 31]. Since trabectedin that is effective against LPSs [10–12] was previously reported to impair HMGA1 activity [19], we treated 402-91 WT and 402-91 ET cells with trabectedin and assessed for the mesenchymal markers. Trabectedin treatment reduced HMGA1, E2F1, ZEB1, and vimentin expression at 24 and 48 h in the sensitive 402-91 WT cells but not in the resistant 402-91 ET cells (Fig. 4a). The RT-PCR data demonstrates that trabectedin treatment regulates the mesenchymal markers of sensitive MLS cells at the transcriptional level and suggests that this regulation might occur through the HMGA1/E2F1 axis (Fig. 4b). Indeed, upon treatment with trabectedin, we found a statistically significant reduction of HMGA1 (*P* < 0.001), E2F1 (*P* < 0.001), ZEB1 (*P* < 0.0001), and vimentin (*P* < 0.0001) mRNA levels in the sensitive 402-91 WT cells (Fig. 4b). At the basal level, resistant 402-91 ET cells express higher levels than WT cells and trabectedin treatment did not induce any significant difference in resistant 402-91 ET cells (Fig. 4b). To confirm HMGA1/E2F1 axis, we interfered with HMGA1 expression in both cells lines and found that transient HMGA1 depletion downregulates E2F1 expression only in 402-91 WT cells (Fig. S5A, and B). Our in vitro results were confirmed in vivo by the use of a PDX model derived from a MLS patient biopsy named ML017, sensitive to trabectedin (#402, #434, and #438) and their counterparts that, following repeated in vivo treatment with trabectedin, became resistant to the drug (#319, #347, and #369). The trabectedin-resistant PDX expressed high level of HMGA1, E2F1, and Vimentin compared with the basal ones found in the sensitive counterparts, which express low to undetectable basal levels (Fig. 4c, d) (*P* < 0.01). The analysis of ZEB1 revealed a mild but not significant increase of the protein in resistant PDX model (Fig. 4c, d). Furthermore, we analyzed specimens derived from tumor bioptic samples of two patients with high-risk MLS who received neo-adjuvant therapy with trabectedin within the clinical trial ISG-STS-10.01 (NCT01710176, EUDRACT 2010-023484-17) [32]. The IHC analysis revealed that the biopsies of both MLSs expressed HMGA1, E2F1, and vimentin that were significantly reduced on the surgical samples obtained after trabectedin therapy and that some remaining cells were negative for the expression of the markers analyzed (Fig. 5, a vs. b and c vs. d) suggesting that trabectedin inhibits the mesenchymal markers also in vivo. Of relevance, we also observed that the treatment caused the translocation of vimentin from the cytoplasm to the nucleus (Fig. 5). The IHC analysis obtained from specimens derived from superior limb and parathyroid metastases of one patient whose tumor had progressed showed an increase of positive cells for HMGA1, E2F1, and vimentin (Fig. 5e, f) supporting the role of HMGA1/E2F1 axis in MLS progression.

### Inhibition of NFkB pathway re-sensitize MLS resistant-cells to trabectedin treatment

**Fig. 6:**
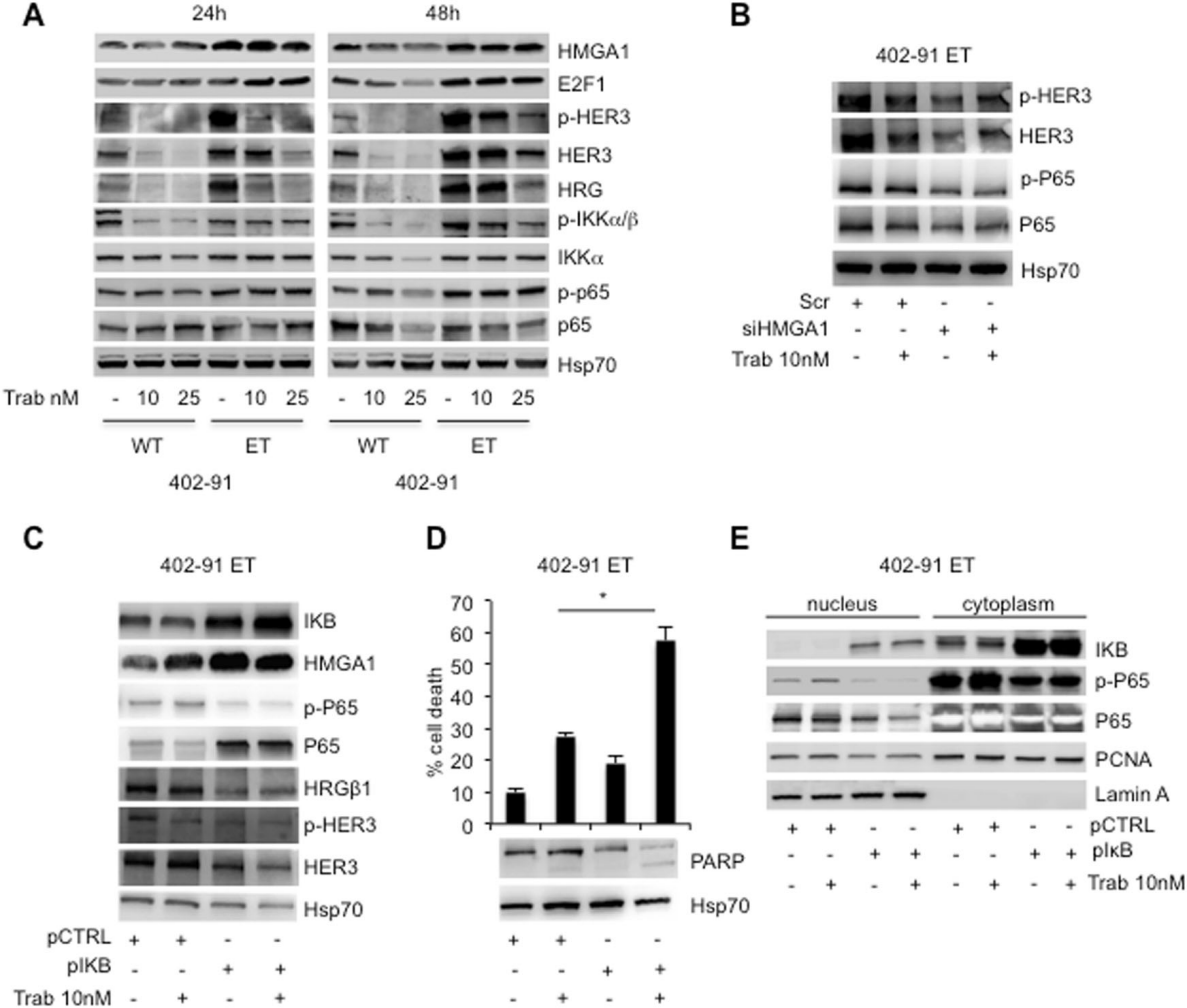
Inhibition of NFkB pathway re-sensitizes MLS resistant-cells to trabectedin treatment. **a** Total cell lysates from 402-91 WT and 402-91 ET cells, upon trabectedin treatment for the indicated time, were evaluated by WB for expression of HMGA1, E2F1, phospho-, and total HER3, HRGß1, phospho-IKKa/b and total IKKa, phospho- and total p65, and Hsp70. **b** Total cell lysates derived from 402-91 ET cells siScr and siHMGA1 untreated or treated cells were analyzed by WB for the expression of phospho- and total HER3, phospho- and total P65 and Hsp70. **c**–**d** 402-91 ET cells were transiently transfected with empty vector or vector containing IKB cDNA, and treated for 48 h with trabectedin. Total cell lysates from controls and transfected cells were analyzed by WB for the expression of IKB, HMGA1, phospho- and total p65, HRGβ1, phospho- and total HER3, Hsp70 and PARP. Cell death was evaluated by Trypan blue exclusion from three independent experiments (*P* < 0.01). **e** Nucleic and cytoplasmic fractions of 402-91 ET cells were analyzed for the expression of IKB, total and phospho-p65, PCNA and LaminA

As above reported, trabectedin treatment downregulated HMGA1 and E2F1 expression in sensitive cells but not in resistant cells (Fig. 4a). In addition, trabectedin treatment induced downregulation of NFkB pathway as revealed by the inhibition of IKKα/β and p65 phosphorylation in sensitive but only at very low extent in resistant cells (Fig. 6a), suggesting that NFkB pathway could be involved in the mechanism of trabectedin-resistance. To confirm this hypothesis, we introduced the NFkB inhibitor IKB in the resistant 402-91 ET cells, to verify whether the inhibition of the pathway re-sensitizes the cells to trabectedin treatment. Overexpression of IKB in these cells caused a significant downregulation of p65 phosphorylation regardless of total protein accumulation (Fig. 6c). The analysis of the nucleus and the cytoplasm fractions revealed the accumulation of phospho-p65 in the cytoplasm confirming the inactivation of the protein (Fig. 6e). Importantly, inhibition of NFkB or trabectedin treatment per se did not induce a statistically significant cell death compared with untreated cells, while their combination re-sensitizes cells to the treatment inducing cell death by apoptosis as revealed by PARP cleavage (Fig. 6d) (*P* < 0.01). In order to elucidate a possible crosstalk between NFkB and HMGA1, we found that IKB overexpression increases HMGA1, suggesting an attempt of the cells to activate a compensatory mechanism of survival (Fig. 6c). Even though the molecular mechanism is not yet clear, the crosstalk between the two pathways is further supported by the finding that siHMGA1 per se inhibits phospho- and total P65 and HER3 (Fig. 6b).

**Fig. 7:**
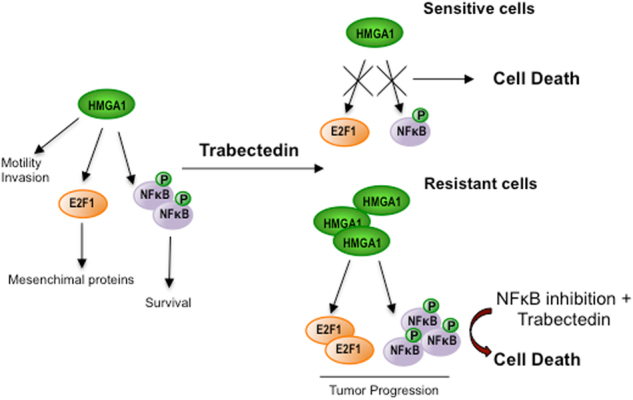
The cross talk between HMGA1 and NFkB pathways. In sensitive cells, trabectedin binding the minor grove of DNA inhibits HMGA1 expression and function that results in cell death. In resistant cells, trabectedin treatment induces increases of HMGA1 expression and NFkB activity that result in tumor progression

It has been previously reported that NFkB binds the promoter of Heregulins (HRGβ1) and activates its transcription [33, 34] that, in turn, activates the EGFR family members signaling on NFkB canonical pathway [35]. Thus, we asked whether HRGβ1/HER3 pathway was involved in the mechanism of drug resistance to trabectedin. We found that trabectedin treatment downregulated HRGβ1 and HER3 expression and phosphorylation in sensitive cells but not in resistant cells (Fig. 6a). Moreover, the overexpression of IKB induced a robust reduction of HRGβ1 expression, and of HER3 expression and phosphorylation in ET resistant cells, suggesting their involvement in MLS tumorigeneity. Figure 7 summarizes the complex cross talk between HMGA1 and NFkB pathways.

In conclusion, our studies strongly support the finding that, at least in DDLS and MLS, the HMGA1/E2F1 and NFkB pathways are involved in the mechanism of tumor progression and trabectedin resistance.

## Discussion

### The role HMGA1 in cell motility and invasion

The HMGA non-histone chromatin proteins are implicated through different mechanisms in tumor progression of several solid neoplasms. The HMGA genes are largely expressed during embryogenesis, whereas they are absent or expressed at low level in adult tissues [36]. HMGA2 rearrangements are well known in several mesenchymal tumors, conversely little is known about the expression and function of HMGA1 in these tumors. In this study, we have evaluated the expression of HMGA1 in a cohort of patients surgically treated at our Institute. The significant high expression of HMGA1 protein in DDLS and MLS compared with WDLS, at protein and mRNA levels, suggests a role for this protein at least in these two subtypes of LPS progression. Depletion of HMGA1 in DDLS and MLS cell lines expressing high level of the protein causes a strong reduction in cell proliferation, induces apoptosis, impairs cell motility and invasion. Furthermore, depletion of HMGA1 in MLS trabectedin-resistant cells restores responsiveness to trabectedin treatment demonstrating for the first time a specific role of HMGA1 in LPS progression and pharmacological resistance. On the contrary, the high sensitivity of 402-91 WT cells to the drug did not revealed any significant additive effect upon HMGA1 depletion. This result suggests that HMGA1 expression in sensitive cells is higher involved in the mechanism of cell motility and invasion rather then drug resistance.

### Trabectedin affects HMGA1/E2F1 axis

One of the mechanisms of action of trabectedin is the transcriptional inhibition of specific genes such as cytokines, chemokynes, and growth factors [29, 37]. Indeed, it has been found that trabectedin impairs the function of HMGA proteins reducing their binding to its responsive promoters causing an increase of cell toxicity compared with cells not expressing HMGA proteins [19]. In agreement with these data, we found that trabectedin treatment downregulates, at the transcriptional level, HMGA1 in trabectedin-sensitive MLS cells but not in the trabectedin-resistant ones. The molecular mechanism by which trabectedin regulates HMGA1 expression in resistant cells is still unclear. In line with data reported in other tumors, we hypothesized that, in response to DNA damage, there is an increase of ATM that, in turn, induces HMGA1 transcription [38]. However, we could not confirm this hypothesis in our resistant 402-91 ET cells.

The downregulation of E2F1 transcription in trabectedin-sensitive MLS cells could be explained assuming that trabectedin inhibits the binding between HMGA1 and E2F1 [19]. Since it has been demonstrated that E2F1 interacts with a 193 bp region of the HMGA1 promoter modulating its transcription [39] we can also assume that the inhibition of HMGA1 is the consequence of downregulation of E2F1 or that trabectedin causes a negative loop in which the two transcription factors regulate each other. Our hypothesis is reinforced by previous findings demonstrating that, even though at not physiological concentration of the drug (between 30 and 500 μM), it has been demonstrated that trabectedin reduces the binding of E2F to its cognate promoter [40]. Indeed, we found that the inhibition of E2F1 in turn inhibits in vitro the mesenchymal markers expression. These results were further confirmed in vivo in a PDX model derived MLS sensitive cells and their counterpart resistant to trabectedin, and in specimens derived from patients with MLS receiving trabectedin as neoadjuvant therapy. In human specimens, the downregulation of HMGA1, E2F1, and vimentin, analyzed by IHC, compared with the basal levels found in the biopsies revealed that trabectedin therapy reduces the mesenchymal markers of the tumor. The translocation of vimentin to the nuclei up on trabectedin treatment suggests that the tumors under treatment could select highly aggressive cells in keeping with previous reports in nasopharyngeal carcinoma where the nuclear vimentin expression correlates with lower survival [41]. In agreement the IHC analysis of specimens derived from two recurrences in different sites of the body of the second patient revealed translocation of vimentin to the nuclei and expression of HMGA1 and E2F1 to the levels found before neo-adjuvant therapy. These results confirm that cell populations with a more aggressive phenotype could be selected during the neoadjuvant therapy; in these cells activation of the HMGA1/E2F1 axis could regulates, at least in part, the progression of these tumors.

### Cross talk between HMGA1 and NFkB pathways

The downregulation of HER3 phosphorylation upon trabectedin treatment in sensitive but not in resistant cells revealed a possible involvement of HER3 in the mechanism of drug resistance as it was previously found in other tumors [42–45]. Since it has been previously described in breast cancer a cross talk between NFkB pathway and ErbB receptors in modulating carcinogenesis we verified whether this cross talk occurs also in LPS [35]. The downregulation of NFkB pathway upon trabectedin treatment in sensitive but not in resistant cells revealed a new mechanism of drug resistance in LPS. Indeed, in MLS sensitive cells we found a strong downregulation of p65 and IKKα/β phosphorylation as well as HER3 and HRGβ1 following trabectedin treatment, suggesting a link between the two pathways. In agreement, it has been described that HRGβ1 promoter contains responsive element for NFkB transcription factor [33], thus suggesting that both pathways contribute to trabectedin resistance through a mechanism leaded by NFkB. In resistant cells, trabectedin does not cause any effect and only the inhibition of NFkB, by IKB, partially restores their responsiveness to trabectedin treatment. The cross talk between HMGA1 and NFkB is revealed by the finding that HMGA1 depletion in resistant cells inhibits P65 while the inhibition of NFkB increases HMGA1 expression suggesting an attempt of the cells to induce compensatory mechanism of survival.

In conclusion, we have identified HMGA1 as a new biomarker of LPS progression and that NFkB pathway contributes to the mechanism of drug resistance in these tumors suggesting a possible combined therapy with NFkB inhibitors and trabectedin to apply for unresponsive LPSs.

## Materials and methods

### Cell lines and transfection

The HF, the thyroid carcinoma cell line 8305c, the LPS cell line SW872 were obtained from Cellosaurus (Amos Bairoch of the CALIPHO group- Swiss Institute of Bioinformatics) and maintained in DMEM medium (Life technologies, Milan, Italy). The myxoid sarcoma cell lines, 402-91 WT and 402-91 ET were produced and maintained as previously described [46, 47].

The SW872 and 402-91 ET were transfected with specific siRNA for HMGA1 (siHMGA1 and siHMGA1 (2) or scramble (siScr) by INTERFERin siRNA Transfection Reagent (Polyplus-transfection, Illkirch, France).

Template oligonucleotides sequences were:

HMGA1: 5′-AAGTGCCAACACCTAAGAGACCCTGTCTC-3′

5′-AAGTCTCTTAGGTGTTGGCACCCTGTCTC-3′

HMGA1 (2): 5′-AAGACCCGGAAAACCACCACACCTGTCTC-3′

5′-AATGTGGTGGTTTTCCGGGTCCCTGTCTC-3′

Scr: -5′AAGCGCAACTCTACCTCTACCTGTCTC-3′

5′-AATAGAGGTAGAGTTGCGCGCCCTGTCTC-3′

The 402-91 WT and 402-91 ET were transfected with pIRES-HMGA1 expression vector (Origene, Rockville, MD, USA) and with pSK-IKBmut by jetPRIME DNA Transfection Reagent (Polyplus-transfection, Illkirch, France).

### Drug and treatments

Trabectedin kindly provided by PhamaMar (PharmaMar S.A., Colmenar Viejo, Spain) was stocked in DMSO at a concentration of 1 mM and stored at −20 °C. The drug was diluted in RPMI medium before treatment at the final concentration of 10 and 25 nM for 24 and 48 h.

### Antibodies

Anti-HMGA1 (ab129153 and ab4078, Abcam, Cambridge, UK), anti-E2F1, anti-Lamin A and anti-PCNA (KH-95, H-102 and PC-10 Santa Cruz Biotechnology, CA, USA), anti-PARP (#9548), anti-Zeb1 (#3396), anti-Vimentin (#5741), anti-pHER3 (#4791), anti-HER3 (# 12708), anti-HRGβ1 (#2573), anti-pIKKα/β (#2697), anti-IKKα (#11930), anti-pP65 (#3033), anti-P65 (#8242), anti-IKB (#4814) were from Cell Signaling (Danvers, USA), and anti-Hsp-70 (ab-83392) was from Immunological Sciences (Rome, Italy). HRP-conjugated secondary antibodies were from Bio-Rad (CA, USA).

### Western blot analysis

All cell lines, before and after transfection and/or treatment, were lysed, analyzed by SDS-PAGE, transferred to nitrocellulose (BioRad) and probed (WB) with antibodies of interest and secondary HRP-conjugated antibodies as previously descrbed [48].

### Semi-quantitative and quantitative RT-PCR

Total RNA from HF, 8305c, SW872, 402-91 WT, and 402-91 ET was prepared using TRIzol® (Ambion). First-strand cDNA was synthesized with the M-MLV RT kit (Invitrogen, Glasgow, UK). Human tissue samples were obtained from the Regina Elena National Cancer Institute, after approval by the institutional ethic committee. Total RNA extraction and RT-PCR were performed as previously described [49].

Primer sequences used to perform semi-quantitative PCR were:

HMGA1: Fw-5′TAGGGAGTCAGAAGGAGCCC, Rev-5′CTGCTCCTCCTCCGAGGAC

Aldolase: Fw-5′CGCAGAAGGGGTCCTGGTGA, Rev 5′CAGCTCCTTCTTCTGCTGCTCCG GGGT

Primer sequences to perform qPCR were:

HMGA1: Fw-5′AAGACCCGGAAAACCACCAC, Rev-5′GCCCTCCTCTTCCTCCTTCT

E2F1 Fw-5′CCCATCCCAGGAGGTCACTT, Rev-5′CTGCAGGCTCACTGCTCTC

VIMENTIN Fw-5′CGCCAACTACATCGACAAGGTGC, Rev-5′CTGGTCCACCTGCCGGCGCAG

ZEB1 Fw-5′AGCAGTGAAAGAGAAGGGAATGC, Rev-5′GGTCCTCTTCAGGTGCCTCAG

GAPDH: Fw-5′TCCCTGAGCTGAACGGGAAG, Rev-5′GGAGGAGTGGGTGTCGCTGT

### Cohort of patients and immunohistochemistry

Formalin-fixed paraffin-embedded sections, from the cohort of 68 patients with LPS surgically treated at the Regina Elena National Cancer Institute (16 DDLS, 12 PLS, 25 MLS, 15 WDLS), were analyzed. The pathologist confirmed by RT-PCR and Fish all karyotype disorders. Furthermore, were analyzed: (i) specimens derived from MLS of two patients with high-risk who received trabectedin treatment as neo-adjuvant chemotherapy (trabectedin 1·3 mg/m^2^ via 24-h continuous infusion, repeated every 21 days) in ISG-STS-10-01 trial (NCT01710176, EUDRACT 2010-023484-17) [32]; (ii) specimens of the superior limb and paratiroid metastases (appeared 1 and 2 years after neo-adjuvant chemotherapy, respectively) derived from one of the patient who MLS has progressed. The IHC was perfomed as previously described [49]. The study was reviewed and approved by the ethical committee of Regina Elena National Cancer Institute, and informed consent was obtained from all patients.

### Cell migration and invasion assays

Chemotaxis and chemoinvasion assays were performed by the use of SW872, 402-91 WT, and 402-91 ET cells upon HMGA1 depletion or overexpression, 24 or 48 h post-transfection.

Chemotaxis and chemoinvasion assays were assessed using a 48-well modified Boyden’s chamber (NeuroProbe, Pleasanton, CA) and 8-mm pore polyvinylpyrrolidone–free polycarbonate Nucleopore filters (Costar, New York, USA). The lower compartment of the chamber was filled with conditioned serum free medium produced from NIH3T3 fibroblasts. The cells were placed in the upper compartment of the Boyden’s chamber. To perform chemoinvasion assay the filters were pre-coated with 20 μg/ml Matrigel (BD Biosciences, Milan, Italy). After 6 or 8 h of incubation at 37 °C, migrated or invaded cells on the lower surface of the filters were fixed, stained with DiffQuick (Merz-Dade, Dudingen, Switzerland) and counted. Each assay was carried out in quadruplicate and repeated at least three times. The ability of the cells to adhere to the filters was verified by staining the upper side of the filter for each cell line.

Images were obtained by Microscope OLYMPUS BX53; scale bars = 20 µm.

### Cell vitality and apoptosis

SW872, 402-91 WT, and 402-91 ET cells parental and/or transient transfected with specific siHMGA using a short exposure were treated for 1 h with trabectedin at a concentration of 10 and 25. Thus, cell vitality was evaluated by Trypan blue exclusion 24 and 48 h after removal of the drug.

For distinguishing apoptotic and necrotic cells, adherent cells were stained with Annexin V-FITC or and propidium iodide (PI) as previuosly described [49].

### Animal studies

Procedures involving animals and their care were conducted in conformity with Italian Governing Law (D.lgs 26/2014; Authorization n.19/2008-A issued March 6, 2008 by Ministry of Health); Mario Negri Institutional Regulations and Policies provided internal authorization (Quality Management System Certificate – UNI EN ISO 9001:2008 – Reg. N° 6121); the NIH Guide for the Care and Use of Laboratory Animals (2011 edition) and EU directives and guidelines (EEC Council Directive 2010/63/UE) and in line with Guidelines for the welfare and use of animals in cancer research (Workman, 2010). Animal experiments were performed as previously described [50].

### Statistical analysis

The IHC levels of HMGA1 expression were scored semi-quantitatively based on staining intensity and distribution percentage using the immune-reactive score (IRS, staining intensity × percentage of positive cells).

Data were reported as mean and standard deviation. Differences were considered statistically significant when *P* ≤ 0.05. Student's *t* test was performed for the comparison of results from qRT-PCR and from all other different test (*P < 0.05, **P < 0.001, ***P < 0.0001).

## Electronic supplementary material

Supplementary figure legends

Figure S1

Figure S2

Figure S3

Figure S4

Figure S5
