# Supplementary material for: HMGA1/E2F1 axis and NFkB pathways regulate LPS progression and trabectedin resistance
Source: Oncogene. 2018 Jul 6;37(45):5926–38. doi: 10.1038/s41388-018-0394-x (PMC6224401; doi:10.1038/s41388-018-0394-x)
Supplement: Supplementary file 1 — Supplementary figure legends [file 41388_2018_394_MOESM1_ESM.docx]

Supplementary figure legends

Figure S1. HMGA1 is highly expressed in liposarcoma cell line SW872. HMGA1 expression was analyzed by WB and PCR in sarcoma cell line SW872. HF and 8305c cells were used as negative and positive internal control, as well as aldolase.

**Figure S2. Depletion of HMGA1 inhibits cell proliferation, induces cell death, and impairs *in vitro* motility and invasion of SW872 LPS cells.** (A) Total cell lysates from HMGA1-depleted cells with siHMGA (2) were analyzed by WB. (B) Cell viability was evaluated by Trypan blue exclusion from three independent experiments (p<0.001). (C) Chemotaxis and (D) chemoinvasion assays were performed and the percentage of migrated and invading cells was presented as the means ± SD of triplicate experiments (p<0.0001) (upper panels). Representative images derived from the same experiments were reported (lower panels).

**Figure S3. Overexpression of HMGA1 in MLS cells promotes *in vitro* motility and invasion*.*** (A) Total cell lysates from 402-91 WT and 402-91 ET cells ectopically transfected with pcDNA expression vector containing HMGA1 cDNA were analyzed by WB for the expression of HMGA1 and normalized for the expression of Hsp70 protein. (B) Chemotaxis and (C) chemoinvasion assays were performed and the percentage of migrated and invading cells was presented as the means ± SD of triplicate experiments (p<0.0001). Representative images of the same experiments were reported (lower panels).

**Figure S4.** **Trabectedin treatment down-regulates NFkB pathway and induces apoptosis of myxoid liposarcoma cells.** (A) Apoptosis, at 24 and 48 h upon trabectedin treatment, **ER3-dependentHER3-dependent**

was evaluated by Annexin-V/PI flow cytometric assay following standard procedures. Representative scatter plots of PI *vs* Annexin V is reported. (B) Cell death of trabectedin treated *vs* untreated cells was evaluated by Trypan blue exclusion (at 24 h p<0.01 and 48 h p<0.001, respectively).

**Figure S5.** (A) Total cell lysates derived from siScr and siHMGA1 untreated or treated cells were analyzed by WB for the expression of E2F1, vimentin, ZEB1 and Hsp70. (B) Relative expression was quantified by ImageJ 1.47v using Hsp70 protein for normalization.
