# Supplementary figures and images for: HMGA1/E2F1 axis and NFkB pathways regulate LPS progression and trabectedin resistance
Source: Oncogene. 2018 Jul 6;37(45):5926–38. doi: 10.1038/s41388-018-0394-x (PMC6224401; doi:10.1038/s41388-018-0394-x)

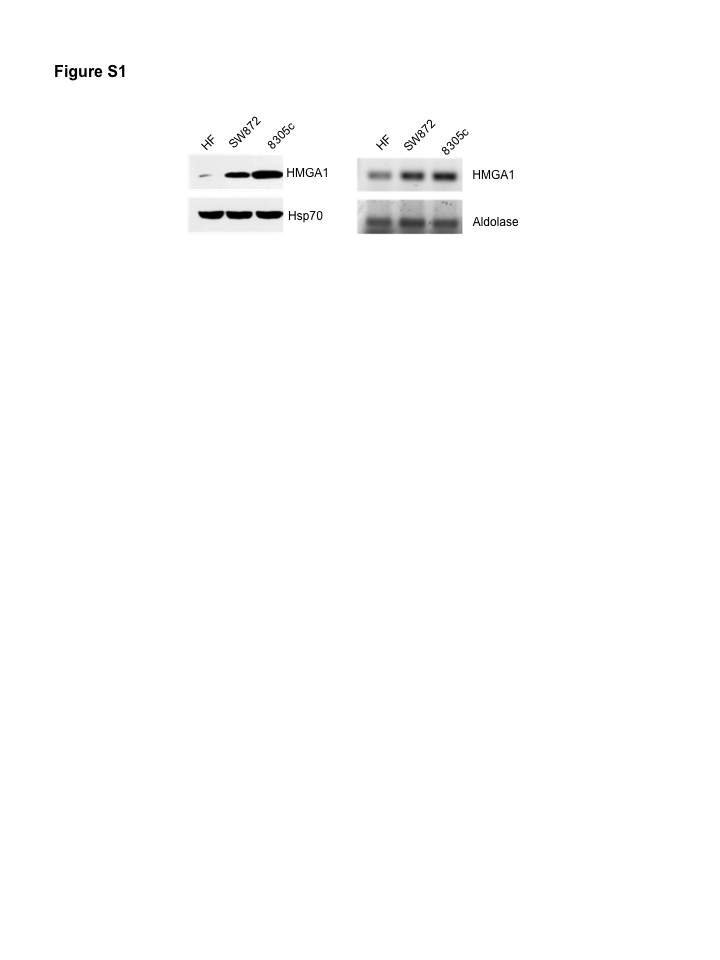

Supplement: Supplementary file 2 — Figure S1 [file 41388_2018_394_MOESM2_ESM.tif]

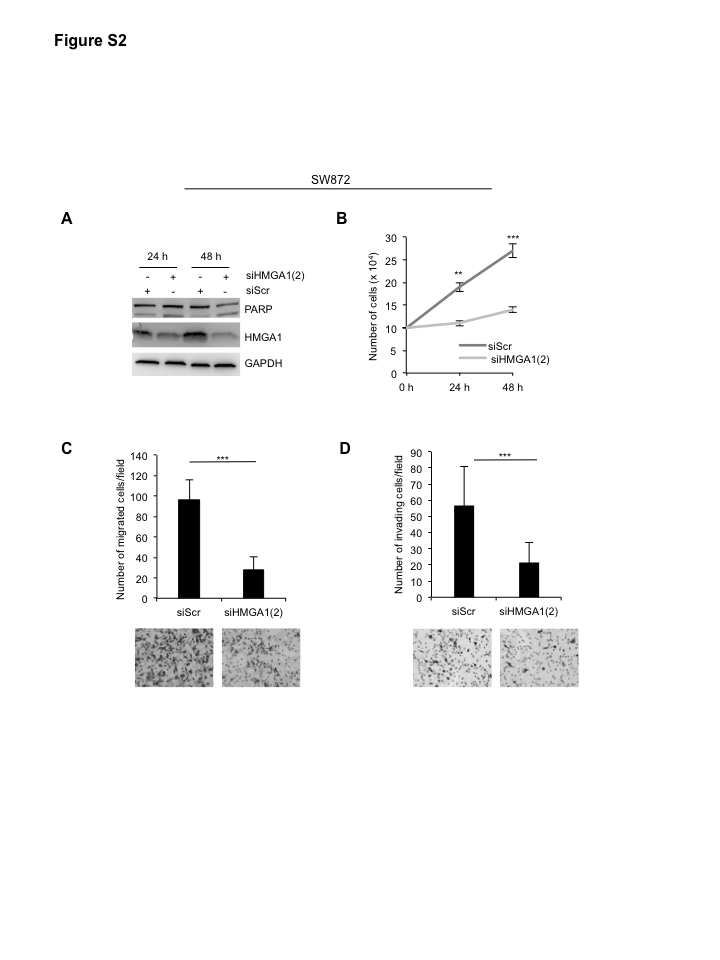

Supplement: Supplementary file 3 — Figure S2 [file 41388_2018_394_MOESM3_ESM.tif]

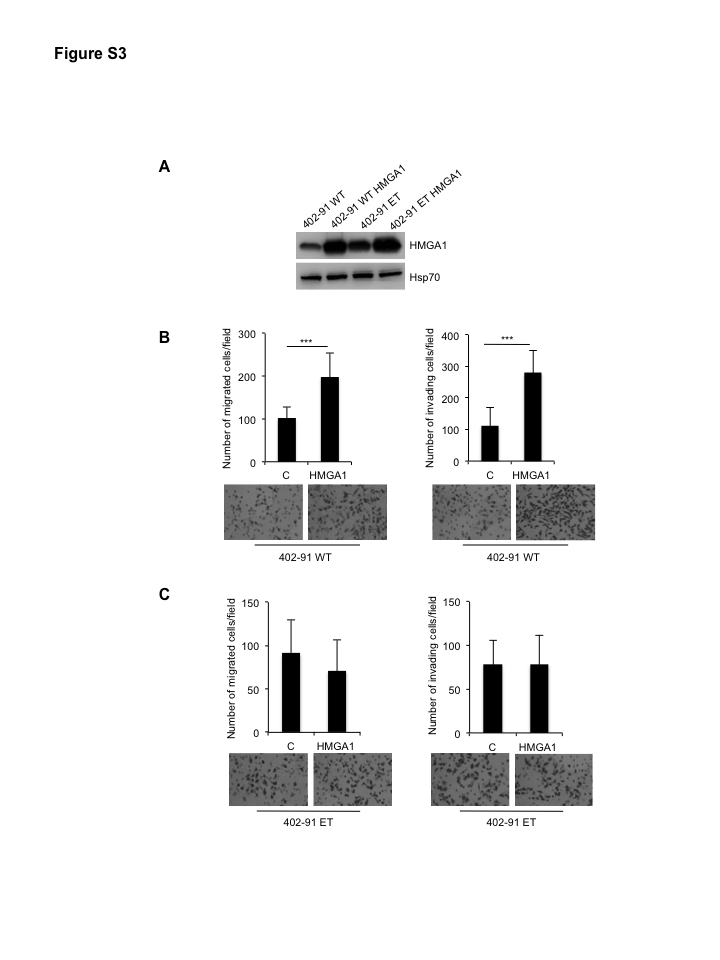

Supplement: Supplementary file 4 — Figure S3 [file 41388_2018_394_MOESM4_ESM.tif]

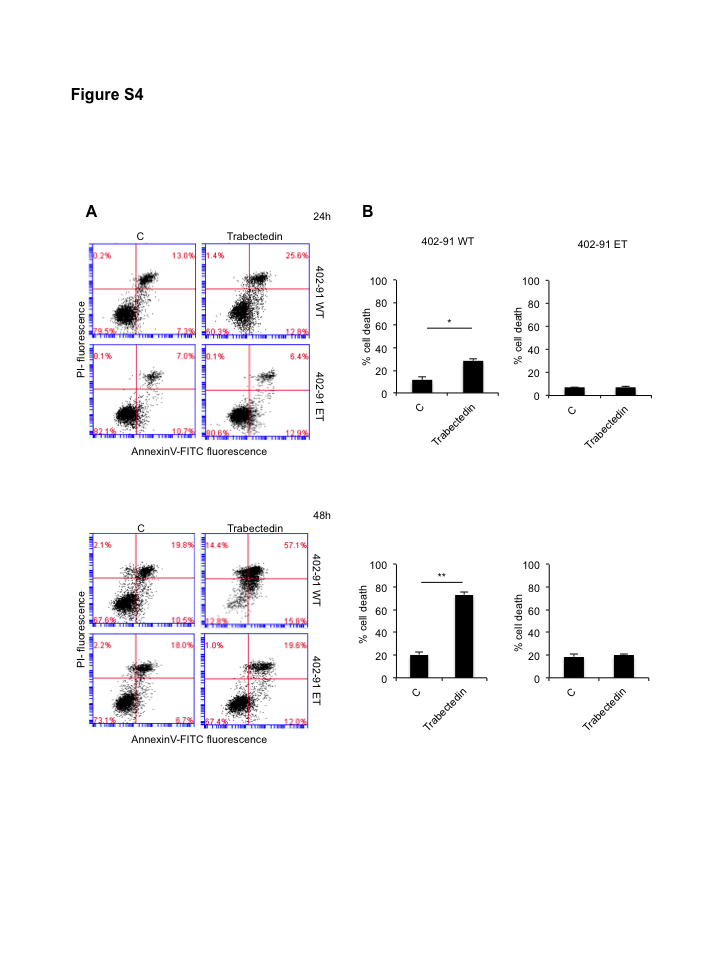

Supplement: Supplementary file 5 — Figure S4 [file 41388_2018_394_MOESM5_ESM.tif]

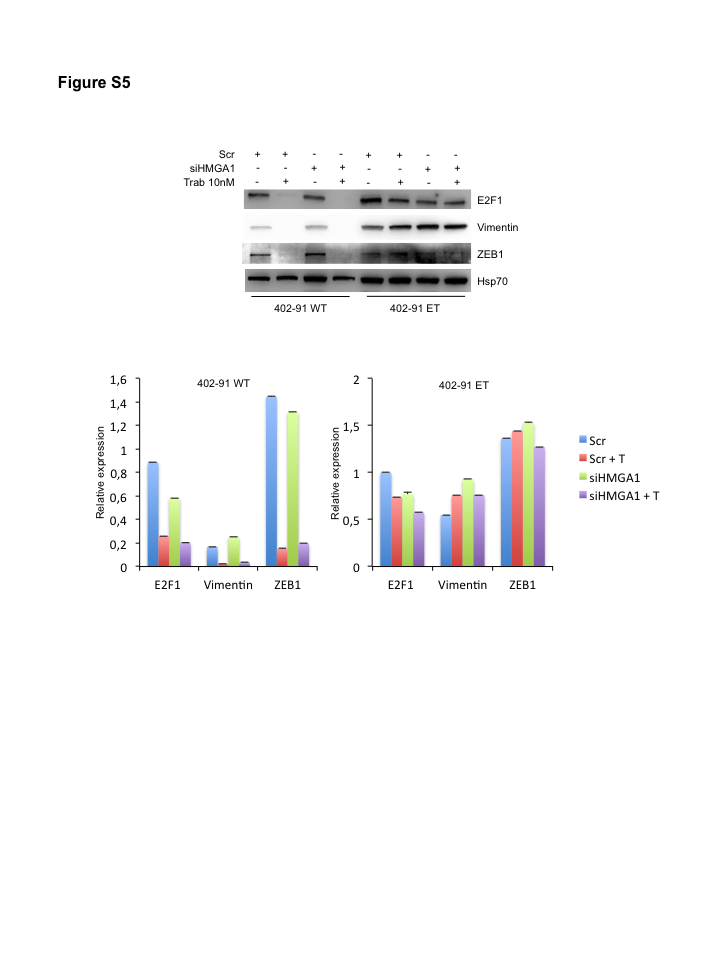

Supplement: Supplementary file 6 — Figure S5 [file 41388_2018_394_MOESM6_ESM.tif]
